# Supplementary material for: A Novel 16S rRNA PCR-Restriction Fragment Length Polymorphism Assay to Accurately Distinguish Zoonotic Capnocytophaga canimorsus and C. cynodegmi
Source: Microbiol Spectr. 2023 May 17;11(3):e02916-22. doi: 10.1128/spectrum.02916-22 (PMC10269634; doi:10.1128/spectrum.02916-22)
Supplement: Supplemental file 2 — Supplemental material. Download spectrum.02916-22-s0002.pdf, PDF file, 0.01 MB [file spectrum.02916-22-s0002.pdf]

Supplementary figure legends

Supplementary Figure 1. Predicted RE PpuMI restriction sites in all published *C. canimorsus* strains. Nucleotide polymorphisms occurred predominantly in the expected RE sequence at positions 279 and 300. Light grey boxes indicate the restriction sites. Dark grey boxes indicate diverse nucleotides.

Supplementary Figure 2. Predicted RE KpnI restriction sites in all published *C. cynodegmi* strains. The nucleotide polymorphism that occurred only in the expected RE sequence was at position 475 as a deletion. The light grey box indicates the RE restriction site. The dark grey box indicates diverse nucleotides.
